# P-1640. Fatigue in long COVID: is it influenced by neurological symptoms?

**DOI:** 10.1093/ofid/ofaf695.1816

**Published:** 2026-01-11

**Authors:** Michael Somero, Hemang Patel, Nilamben J Mangukia, Ashish Bhargava, Susan Szpunar, Mamta Sharma, Leonard Johnson, Louis Saravoltaz

**Affiliations:** Henry Ford St John Hospital, Detroit, MI; St John Hospital, Detroit, Michigan; Henry Ford St John Hospital, Detroit, MI; Henry Ford St. John Hospital, Grosse Pointe Woods, Michigan; Henry Ford St John Hospital, Detroit, MI; Henry Ford Health System, Grosse Pointe Woods, Michigan; Henry Ford St. John Hospital, Grosse Pointe Woods, Michigan; Henry Ford Health System, Grosse Pointe Woods, Michigan

## Abstract

**Background:**

Fatigue following acute COVID-19 is common and can be significantly disabling. However, little is known about the severity of fatigue in long COVID (LC) and its connection to co-existing depression and cognitive impairment.

**Methods:**

Our prospective study assessed patients at the LC Clinic (LCC) at Henry Ford St. John Hospital in Detroit, MI, using the Fatigue Assessment Scale (FAS). On their first visit to LCC, patients completed a standardized form documenting their demographics, comorbidities, and symptoms experienced during acute COVID-19 and their symptoms at the time of the visit. Patients were classified by fatigue severity based on FAS scores: mild to moderate (22-34) and severe (over 34). All patients were asked to complete the Patient Health Questionnaire (PHQ)-2, PHQ-9, and the Montreal Cognitive Assessment (MoCA). The assessments from these questionnaires were compared between two cohorts.

**Results:**

Of the 35 patients evaluated, 18 (51.5%) had severe fatigue. The median duration of their first clinic visit to LCC from their acute COVID was 641 days (range=46-1571 days). Self-reported symptoms between these cohorts indicated that shortness of breath (SOB) (p=0.01), chest pain (p=0.05), and feeling down and depressed (p=0.03) from acute COVID were significantly higher among patients with severe fatigue. Similarly, self-reported SOB (p=0.04), dry cough (p=0.02), loss of appetite (p=0.03), headache (p=0.02), and abdominal (p=0.01) and muscle pain (p=0.02) were significantly higher among patients with severe fatigue than those with mild-moderate fatigue. Patients with severe fatigue reported significantly feeling down, depressed, and hopeless (p=0.04), along with little interest and no pleasure in doing things (p=0.01). However, the cognitive impairment assessed by MoCA (p=0.2) did not correlate with the degree of fatigue.

**Conclusion:**

Patients with severe fatigue were more likely to report symptoms like shortness of breath, dry cough, loss of appetite, headaches, and muscle pain than those with mild to moderate fatigue during their first LCC visit. Higher PHQ-2 and PHQ-9 scores, indicating greater neurological symptom burden, were linked to increased fatigue severity, but these symptoms did not significantly impact cognitive impairment in long-haul patients.

**Disclosures:**

All Authors: No reported disclosures

